# Complete Heart Block in the Fetus: An Antenatal Complication of Sjögren’s Syndrome

**DOI:** 10.3390/life15121890

**Published:** 2025-12-11

**Authors:** Maria Videnie, Cristian Viorel Poalelungi, Andreea Chiriac, Anca Bobircă, Maria-Cristina Alexandru, Iuliana Ceaușu

**Affiliations:** 1 Faculty of Medicine, “Carol Davila” University of Medicine and Pharmacy, 020021 Bucharest, Romania; videnie-maria.moraru@drd.umfcd.ro (M.V.); andreea.l.chiriac@rez.umfcd.ro (A.C.); anca.bobirca@umfcd.ro (A.B.); maria-cristina.steopoaie@drd.umfcd.ro (M.-C.A.); iuliana.ceausu@umfcd.ro (I.C.); 2 Department of Obstetrics and Gynaecology, “Dr. I. Cantacuzino” Hospital, 030167 Bucharest, Romania; 3 Department of Rheumatology, “Dr. I. Cantacuzino” Hospital, 030167 Bucharest, Romania

**Keywords:** autoimmune disease, pregnancy, in vitro fertilization, high-risk pregnancy, complete fetal atrioventricular block

## Abstract

**Background:** Complete fetal atrioventricular block (CAVB) is a rare but life-threatening condition, occurring in approximately 1–2% of pregnancies associated with maternal anti-Ro/SSA antibodies. The transplacental migration of anti-Ro/SSA and anti-La/SSB antibodies damages the fetal cardiac system, leading to sustained bradycardia, cardiomyopathy, fetal hydrops, and intrauterine fetal demise. Despite the use of fluorinated corticosteroids or β-agonists, therapeutic efficacy remains limited once a complete block is established. **Case Presentation:** We present the case of a 35-year-old primigravida with a pregnancy achieved through in vitro fertilization (IVF). At 20 weeks of gestation, she was referred to our emergency unit due to persistent fetal bradycardia. Fetal echocardiography confirmed CAVB with a ventricular rate of 64 bpm. Maternal serologic testing was positive for anti-Ro/SSA and anti-La/SSB antibodies, suggesting an autoimmune etiology. Treatment with oral dexamethasone and salbutamol was initiated, but follow-up echocardiography at 24 weeks showed worsening cardiac status, including reduced ventricular rate of 59 bpm, cardiomegaly, and pericardial effusion. Intrauterine fetal death occurred at 25 weeks of gestation. **Management and Outcome:** Four months postpartum, the patient underwent a minor salivary gland biopsy. Histopathological evaluation confirmed the diagnosis of primary Sjögren’s syndrome. **Conclusions:** This case illustrates the severe consequences of autoimmune-mediated CAVB and the limited effectiveness of available treatments once a complete block has developed. It underscores the importance of early fetal rhythm surveillance and targeted maternal autoimmune screening—particularly before assisted reproduction, where structured preconception evaluation offers an opportunity for earlier recognition and risk stratification. Earlier detection may improve counseling and management strategies in future pregnancies.

## 1. Introduction

Complete fetal atrioventricular block (CAVB) is a rare but serious cardiac disorder characterized by complete dissociation between atrial and ventricular activity [1]. Its estimated incidence is about 1 in 15,000 to 20,000 live births [2], and it is associated with considerable fetal and neonatal morbidity and mortality [3]. CAVB may occur in association with congenital structural heart disease or, in structurally normal fetuses, as an immune-mediated condition. In the latter, the most frequent cause is the transplacental transfer of maternal anti-Ro/SSA and anti-La/SSB antibodies [4,5,6,7]. These antibodies cause inflammation and scarring of fetal conduction tissue, particularly the atrioventricular node, leading to irreversible conduction block [8,9,10].

Maternal autoimmune conditions such as systemic lupus erythematosus and Sjögren’s syndrome are commonly associated with immune-mediated congenital AV block [11,12]. Sjögren’s syndrome is strongly linked to the presence of anti-Ro and anti-La antibodies [13,14]. Notably, many affected women remain asymptomatic, and fetal heart block is often the first indication of an undiagnosed autoimmune condition [13,15,16].

Women undergoing in vitro fertilization (IVF) represent a distinct subset of pregnant patients in whom early and thorough preconception assessment is critical. Standard fertility protocols typically do not include autoantibody testing unless autoimmune disease is suspected. However, autoimmune disorders are more frequent among infertile women than fertile women [17], and the hormonal therapy used during IVF could mask underlying autoimmune symptoms. This situation can delay risk identification and reduce the opportunity for preventive strategies, such as serial fetal echocardiography or prophylactic hydroxychloroquine in antibody-positive patients.

This case report describes a pregnancy complicated by immune-mediated CAVB secondary to previously undiagnosed maternal Sjögren’s syndrome. It highlights the importance of early risk stratification, close prenatal monitoring, and comprehensive preconception evaluation—particularly in pregnancies achieved through assisted reproductive technologies, where unrecognized autoimmune disease may result in severe fetal complications.

## 2. Case Presentation

A 35-year-old primigravida with an uneventful in vitro fertilization (IVF) pregnancy was referred at 20 weeks of gestation to the Obstetrics-Gynecology—Fetal Medicine Department at “Dr. I. Cantacuzino” Hospital, following a routine ultrasound control that indicated persistent fetal bradycardia. Standard infertility investigations were completed before IVF, without any suggestive manifestations of autoimmune or thrombotic disorders. Autoimmune antibody screening (including anti-Ro/SSA and anti-La/SSB) was not performed at that time, as the patient was clinically asymptomatic.

**Figure 1:**
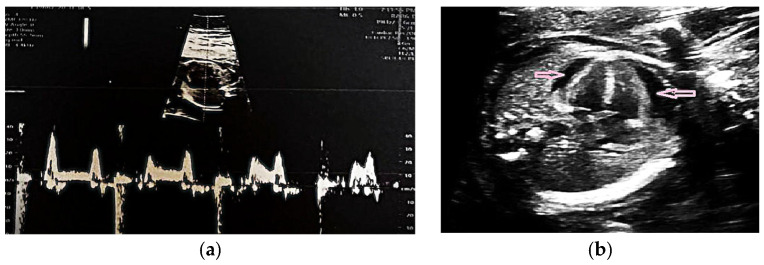
(**a**) Fetal echocardiography at 20 weeks of gestation with CAVB with a heart rate of 64 beats per minute (bpm); (**b**) Fetal echocardiography at 24 weeks of gestation showed a heart rate of 59 bpm, cardiomegaly and pericardial effusion (pink arrows).

Fetal echocardiography confirmed CAVB with a ventricular rate of 64 bpm (Figure 1a). Subsequent maternal serologic evaluation identified anti-Ro/SSA and anti-La/SSB antibody positivity, indicating an immune-mediated etiology. In the absence of systemic manifestations or laboratory findings suggestive of systemic lupus erythematosus, a primary diagnosis of Sjögren’s syndrome was considered.

In this case, maternal testing (ANA, anti-dsDNA, rheumatoid factor, complement levels, inflammatory markers) was normal except for anti-Ro and anti-La positivity. Antiphospholipid antibody testing (lupus anticoagulant, anticardiolipin, and anti-β2-glycoprotein I) was also performed and yielded negative results, confirming the absence of concomitant antiphospholipid syndrome. This finding further supports that the observed fetal conduction abnormality was driven solely by anti-Ro/SSA and anti-La/SSB autoimmunity.

Additional autoantibody testing was not performed, given the absence of systemic features and maternal clinical stability. While the anti-Ro/La–fetal heart block association is well known, this case is notable for the combination of maternal age (35 years), early diagnosis at 20 weeks’ gestation, and fetal CAVB on echocardiography—an association with implications for early prenatal screening and counseling.

Treatment was initiated with oral dexamethasone (4 mg/day) and salbutamol. The medical team evaluated fetal pacemaker placement through invasive intrauterine intervention, but it was considered inappropriate owing to early gestational age, fetal size, and high procedural risk [18,19,20]. Follow-up fetal echocardiography at 24 weeks showed progressive cardiac deterioration, with the ventricular rate decreasing to 59 bpm and echocardiographic evidence of cardiomegaly and pericardial effusion (Figure 1b). Intrauterine fetal demise occurred at 25 weeks.

**Figure 2:**
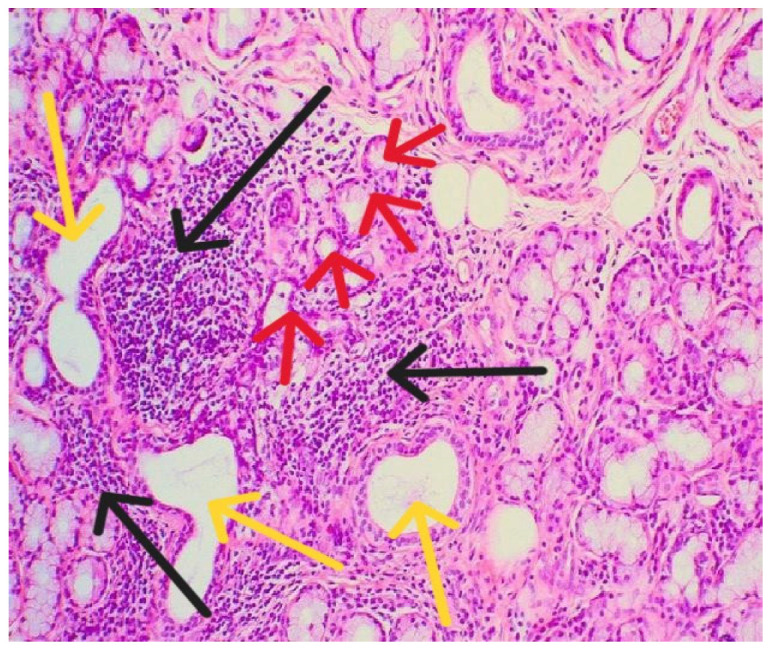
Histopathological examination showed lymphomononuclear cell infiltrates (black arrows) organizing as periductal infiltrates (yellow arrows) in the salivary glands associated with acinar atrophy and destruction (red arrows).

Labor was medically induced, and the maternal postpartum course was uneventful. Four months after delivery, a minor salivary gland biopsy confirmed the diagnosis of primary Sjögren’s syndrome (Figure 2).

This case was conducted as a retrospective analysis. The study received approval from the Institutional Review Board of “Dr. I. Cantacuzino” Hospital (No. 15766/08.04.2024) and adhered to the principles of the Declaration of Helsinki. Written informed consent was obtained from the patient prior to inclusion in this report.

## 3. Discussion

CAVB in fetuses of mothers with Sjögren’s syndrome is primarily immune-mediated and represents the most severe cardiac manifestation of neonatal lupus erythematosus, caused by transplacental passage of maternal IgG autoantibodies [20,21]. From the second trimester onward, increased placental permeability enables anti-Ro/SSA and anti-La/SSB antibodies to enter fetal circulation via FcRn receptors. It coincides with the 18–24-week period when the fetal cardiac conduction system is most vulnerable [22].

Anti-Ro52 antibodies target fetal cardiomyocytes and conduction tissue, including the AV node, and interfere with calcium channel function, triggering apoptosis [15,23,24]. The resulting inflammation leads to fibrosis, permanently blocking atrioventricular conduction [8,25,26,27].

Although exposure to anti-Ro/SSA and anti-La/SSB antibodies is required, most fetuses remain unaffected. Disease expression depends on a combination of maternal and fetal HLA predisposition, antibody specificity, and inflammatory triggers [28,29,30]. Given this multifactorial risk, particularly in asymptomatic women with unexplained infertility undergoing IVF—where underlying autoimmunity may remain undetected—targeted screening may assist risk identification in select IVF candidates.

This case is distinctive because the pregnancy was conceived via IVF, a setting in which autoimmune antibody screening is not routinely performed, particularly in clinically asymptomatic women. The absence of suggestive symptoms prior to fertility treatment allowed underlying autoimmunity to remain undetected, underlining how subclinical Sjögren’s syndrome may escape recognition in standard IVF protocols. As a result, the diagnosis of maternal autoimmunity was made only after the fetal finding of CAVB. This highlights a missed opportunity for earlier risk identification and anticipatory counseling, and reinforces the potential value of targeted autoimmune screening in selected IVF candidates—especially those with unexplained infertility or subtle indicators of immune dysregulation. Integrating such measures into preconception evaluation may improve risk stratification and prenatal surveillance in antibody-positive women.

The case represents a regrettable outcome in an IVF-derived pregnancy, where conventional fertility and antenatal screening failed to detect maternal autoantibodies, ultimately contributing to intrauterine fetal demise. The diagnosis was made at 20 weeks’ gestation in a clinically asymptomatic primigravida. Although causality cannot be confirmed, the absence of targeted screening delayed risk identification and limited the opportunity to implement preventive measures.

### 3.1. Complete Fetal Atrioventricular Block Management

Once complete, CAVB develops, reversal is rare due to established fibrosis of the atrioventricular node. Current management primarily involves maternal corticosteroids and β-agonists to improve fetal hemodynamics [3,6,31,32]. Corticosteroid therapy, usually with dexamethasone, aims to reduce fetal cardiac inflammation in antibody-mediated block; however, its efficacy in complete (third-degree) block remains uncertain. According to the American Heart Association and the European Society of Cardiology, corticosteroids may be considered in fetuses with evolving or second-degree heart block. However, in a complete block, their benefit is unproven and may be associated with fetal growth restriction and oligohydramnios [31,33,34]. Some observational studies suggest potential stabilization when inflammation or myocardial dysfunction is present, provided careful monitoring is possible [35,36,37].

In our case, dexamethasone and salbutamol were initiated after multidisciplinary evaluation. Despite early intervention, the ventricular rate declined and cardiac decompensation progressed, resulting in intrauterine fetal death at 25 weeks. Poor prognostic indicators—including heart rate <60 bpm and cardiac dilation—were already present [34,36,38]. More invasive options, such as in utero pacemaker implantation, remain experimental and have shown high complication rates [37,39].

Hydroxychloroquine (HCQ) has been shown to reduce the risk of immune-mediated CAVB recurrence by approximately 50% when initiated preconceptionally or early in pregnancy. However, the data predominantly involve women with a previous affected pregnancy [40,41]. For future conceptions, early HCQ initiation alongside serial echocardiography between 16 and 26 weeks would be recommended.

Other approaches, such as intravenous immunoglobulin or plasmapheresis, have been explored to reduce maternal antibody load or modulate immune activation. While early reports suggested a possible benefit in incomplete or evolving block, subsequent studies failed to demonstrate consistent improvement in progression or neonatal outcomes [10,32]. Current consensus does not support their routine use outside investigational or highly selected cases [32,42].

### 3.2. Implications for Assisted Reproductive Technologies

This case highlights an important gap in assisted reproductive technologies. Standard IVF protocols typically include screening for infectious diseases, thrombophilia, and basic autoimmune markers such as ANA [43,44]. For IVF patients with unexplained infertility or autoimmune indicators, screening for anti-Ro/SSA and anti-La/SSB antibodies may be considered, as previously outlined. Emerging evidence shows that women with unexplained infertility may harbor silent autoimmunity, and these antibodies have been associated with reduced implantation, impaired pregnancy maintenance, and lower IVF success rates [45,46,47]. Given that autoimmune diseases such as primary Sjögren’s syndrome are increasingly prevalent among reproductive-age women and often remain undiagnosed due to subtle or nonspecific early symptoms [48,49], specific tests could be considered in selected IVF candidates—such as those with unexplained infertility, autoimmune markers, prior adverse pregnancy outcomes, or mild suggestive symptoms.

In this case, maternal Sjögren’s syndrome was identified only after fetal CAVB was diagnosed, highlighting the diagnostic challenge of clinically silent autoimmunity in IVF patients. Incorporating anti-Ro/SSA and anti-La/SSB autoantibody testing into infertility screening and multidisciplinary counseling within IVF protocols permits earlier risk identification, improved prenatal surveillance, and informed planning for future pregnancies.

### 3.3. Sjögren’s Syndrome and Pregnancy

Sjögren’s syndrome is uncommon in pregnancy, which limits clinical experience and complicates management. Pregnancies complicated by anti-Ro/SSA and anti-La/SSB antibodies require special attention due to the risk of fetal atrioventricular block. Although the patient’s ANA test was negative, this does not exclude clinically relevant autoimmunity, as anti-Ro/La antibodies may be detected by solid-phase assays even when ANA by immunofluorescence is negative. This ANA-negative, anti-Ro/La-positive profile is relatively common in asymptomatic or early Sjögren’s syndrome and has been associated with neonatal lupus and congenital AV block [13,14,15,20].

When pregnancy occurs in a mother with known or suspected Sjögren’s syndrome—or when antibody positivity is detected—close fetal surveillance is essential. Serial fetal echocardiography from 16 to 26 weeks is recommended, as this period represents peak vulnerability to immune-mediated conduction abnormalities [50]. Weekly monitoring is advised between 16 and 26 weeks, followed by biweekly evaluations until 34 weeks, to detect early conduction changes that may progress rapidly, sometimes within 24 h [42].

Following diagnosis, the patient was referred for rheumatology follow-up and counseling regarding future pregnancies. In the event of a subsequent conception, hydroxychloroquine prophylaxis (400 mg/day) would be recommended starting preconceptionally or early in gestation, based on data demonstrating a reduction in recurrence of congenital heart block from approximately 18% to 7% in anti-Ro/SSA–positive women [40]. Serial fetal rhythm monitoring during the critical period of antibody activity (16–26 weeks) remains the cornerstone of surveillance. Beyond 26 weeks, monitoring frequency may be reduced if no abnormalities are detected. The patient remains clinically stable under rheumatologic supervision.

## 4. Conclusions

This case highlights the importance of comprehensive preconception evaluation in patients undergoing ART, particularly IVF. Subclinical autoimmunity in otherwise asymptomatic women may contribute to important fetal complications, including irreversible atrioventricular block. Current fetal therapies—such as corticosteroids and β-agonists—may offer temporary improvement but show variable effectiveness and carry potential maternal and fetal risks. In contrast, hydroxychloroquine prophylaxis, particularly when initiated preconceptionally or early in pregnancy, has demonstrated protective benefit in high-risk women, although it remains underutilized. Screening in selected IVF patients could enable earlier risk recognition and guidance for future pregnancies. Given the rarity of Sjögren’s syndrome in pregnancy and the absence of standardized management guidelines, close multidisciplinary follow-up is essential. Serial fetal echocardiography and individualized management based on disease activity and cardiac progression remain the cornerstones of care in antibody-positive pregnancies.

## Data Availability

The original contributions presented in the study are included in the article, further inquiries can be directed to the corresponding author.
